# The mLOS Open-Source Software for Analyzing Length of Stay in Animal Shelters

**DOI:** 10.3390/ani16203207

**Published:** 2026-10-12

**Authors:** Michael Loizos Mavrovouniotis

**Affiliations:** Social Compassion in Legislation, P.O. Box 1125, Laguna Beach, CA 92652-1125, USA; mlmavro@yahoo.com

**Keywords:** animal sheltering, length of stay, shelter census, days in care, conditional outcome, conditional length of stay, Kaplan-Meier estimator, Aalen-Johansen estimator, Cox proportional hazards, Weibull regression, cumulative incidence, shelter outcome

## Abstract

Length of stay (LOS) is critical in shelter management and animal welfare. Its assessment by calendar period must account for animals whose stay overlaps with period boundaries. mLOS is an open-source R tool that uses animal-level intake and outcome records to analyze LOS distributions and competing outcomes using survival-analysis methods. A companion Python package, ShelterDataPrep, prepares raw data extracts for analysis. The factors used are calendar period, intake type, and a user-defined animal group. The counting-process formulations left-truncate stays in progress at the beginning of a period and right-censor stays ongoing at period end. Kaplan-Meier estimates the stay distribution, Aalen-Johansen gives cumulative incidence by outcome type, and Cox and Weibull regressions compute the effect of each factor adjusted for others. A run computes in-care stay curves, remaining stay curves, competing outcomes, outcome conditional on tenure, and population-load figures including census. Each is produced for the unified sample and separately by factor. Pooled and per-factor Cox regressions produce hazard ratios while corresponding Weibull regressions produce LOS ratios. Validation includes hand-derived exact tests and simulation fixtures that recover known behavior. The outputs comprise plots, companion CSVs, a JSON with complete results, a consolidated workbook, and an experimental slide deck.

## Simple Summary

Length of stay (LOS) affects animal welfare and shelter resources, but its historical computation is misleading, because it misattributes stays that straddle the start or end of a period. The mLOS open-source software reads a shelter’s intake and outcome records and measures LOS correctly by period, including animals that arrived before the period started or were still in care when it ended. mLOS computes the fraction of animals remaining in care by day after intake; the tenure (elapsed days of care) distribution of residents; and their expected remaining stay and outcome probabilities. mLOS also compares LOS across factors like animal size, intake type, and calendar period, adjusting each for the others. Another tool, ShelterDataPrep, converts shelter records to the right format. In an example (Orange County, California), large dogs were 38% of intakes but about 70% of the dogs in care, their expected future stay growing with their tenure. Stays grew longer from mid-2021 through September 2022, mainly among the longest-staying dogs, without a change in median stay. Shelters can use mLOS to track their own statistics, compare them to those of other shelters or published studies, and explore how policies and programs may impact LOS and census.

## 1. Introduction

Length of stay (LOS) in animal shelters is critical for managing shelter capacity, improving animal welfare, and assessing and improving shelter performance [1,2,3,4,5,6,7,8]. Accordingly, assessment of LOS by calendar period is advisable [9]. The historically used computation of LOS, labeled HistLOS [10,11], is based solely on animals that exit the shelter in a given calendar period. HistLOS computes a period’s LOS using the whole intake-to-outcome stay of every animal whose outcome falls in the period of interest; the stay is counted from intake even if most of the stay occurred before the period of interest; the stay is not counted at all if the animal was still in care at the end of the period. HistLOS is misleading, as it does not properly account for animals whose stay overlaps with the beginning or the end of the period being studied [11]. Misattribution of the stay across periods can make a slow period appear fast and a fast one appear slow. The HistLOS approach appears to be used in shelter software and nationally reported statistics [11]. A separate, parallel issue is that many shelters only compute average (mean or median) LOS, even though the computation of the full LOS distribution may yield additional insights and avert misinterpretation.

LOS can be studied using statistical techniques from the field of survival analysis [12,13] including Kaplan-Meier (KM) time-to-event [14] fits and Cox proportional hazards regressions [15]. The statistical term “survival” here simply denotes residency in the shelter, rather than a specific type of outcome. In survival (time-to-event) analysis, the problems caused by mishandling stays overlapping the beginning or end of a study period (as HistLOS does) are well understood [16] as are the remedies.

A new methodology showed how LOS distributions (labeled ExitLOS) [11] can be derived by calendar period with correct treatment of the overlap of each animal’s stay with the period of interest. The methodology accounts for animals already present at the beginning of a period via left-truncation; and for animals continuing in care past the end of the period via right-censoring [16].

Truncation and censoring matter more when period duration is comparable to the length of animal stays, including the long-stay tail of the distribution. Comparison of one shelter in two consecutive months in 2023 showed a dramatic reversal between HistLOS and ExitLOS [11]. In other examples, the new methodology showed that a shelter’s LOS may exhibit significant change over time, which justifies the rigorous computation of LOS within each period. The change persists even after accounting for the mix of incoming animal sizes and ages, either by stratifying [11] or by including them as predictors in the Cox regression [10].

The methodology was subsequently reimplemented [17] relying on standardized files for data and settings to allow application to any shelter, moving to R’s survival package [18] for more statistical capabilities including Aalen-Johansen (AJ) [19] estimation of cumulative incidence functions (CIF) by outcome type. The open-source software system mLOS is now publicly available in a repository [20]. A small companion open-source package, ShelterDataPrep [21], assists with filtering raw shelter data to convert them into input data for mLOS.

Software is accorded the same importance in the modern scholarly record as other research products [22]. There is no comparable pre-existing software for animal sheltering, and the introduction and use of the software has the potential to alter how shelters analyze their operations, moving them towards data-centered decisions. Easy access to a broad set of metrics enhances the application of any other research result in a shelter. Accordingly, this article outlines the capabilities of this open-source software and explains its use in the detailed analysis of LOS and related metrics. Its presentation here will aid in generating interest and planning future activity: additional statistical capabilities, integration into shelter databases, or interactive user-friendly hosted platforms.

There are similarities among shelters, but also differences. Unaware of their own statistical metrics, shelters may be hesitant to structure their policies based on studies of other shelters. mLOS seeks to make many assertions (related to LOS, outcomes, and census) measurable. The goal is for a shelter to be able to ascertain its own metrics and compare them to those of other shelters or those published in studies, facilitating the adoption of applicable ideas from other shelters and findings from the literature.

Shelter databases are currently geared towards an accounting view of animal data. The tools presented here are part of an emerging view of shelters as complex systems whose operation and decision-making can be improved via stochastic models [4,8], analysis of trends across shelters [23,24], and the detailed animal-level data analysis that is the subject of this article. The role of mLOS is to allow a shelter to determine how its LOS varies over time, across animal groups, or after a policy change; how expected LOS and outcomes change during an animal’s stay; whether outcome types occur at different rates; the composition and tenure distribution of the population in care; and differences in all these metrics by intake type or another grouping. While averages are useful, mLOS usually provides a full curve or distribution, giving shelters opportunities to use informal insights and exercise their own judgment.

The objective of this Technical Note is to present mLOS as open-source software for the analysis of LOS by calendar period, with left-truncation and right-censoring applied at period boundaries. It describes the statistical methods the software implements (Kaplan-Meier, Aalen-Johansen, Cox, and Weibull, together with census and animal-day metrics derived from them), the structure of its inputs and outputs, and its validation. The software is a toolkit rather than a mandated set of metrics, and its target audience is practitioners and their collaborators or advisors. A worked example on dogs at Orange County Animal Care (2019–2022) illustrates a subset of the outputs and their interpretation.

## 2. Materials and Methods

This section is divided into two parts. The first part presents the statistical methods underlying the methodology. The second part presents the software design.

### 2.1. Statistical Methods

Standard time-to-event (survival) analysis estimates the conditional probability of an outcome on day X (counting from intake), among animals still in care, as the outcomes that occur on day X divided by the population at-risk on day X. In LOS by calendar period, this same estimation is narrowed to outcomes and at-risk counts within the specified calendar period; this is the truncation and censoring mechanism described below (Section 2.1.1). In a sequence of adjacent periods, every outcome and at-risk instance is counted in precisely one period. Chaining the daily probabilities produces the probability that a stay exceeds X, the KM survival curve described in Section 2.1.2.

The formal definition of calendar-period LOS, with the decomposition of each stay into one row per period, the entry and exit times of each row on the days-since-intake scale, and its event indicator, is given in §3 of the mLOS math methods document. (Hereafter, § denotes sections of the math methods document.)

The first presentation of this methodology [11] contrasted this approach (labeled ExitLOS there) to the historical approach (HistLOS) and demonstrated that LOS by calendar period matters. Its examples showed that the historical approach is misleading; that statistically significant changes of LOS actually occur by calendar period; and that changes cannot be explained by animal features and are therefore relevant for shelter policy decisions.

#### 2.1.1. Truncation and Censoring

mLOS uses the counting-process formulation of survival analysis, which correctly handles animals with stays overlapping a period boundary. Left-truncation: animals already in care when a period begins are observed only from the period start, not from their intake date, which avoids bias [25]. Right-censoring: animals still in care at the end of a period, or whose stays are capped, are treated as censored rather than excluded. Animals participate in multiple periods if they were in care across period boundaries. (See the first schematic in PLOS One [11].) The math methods document §3 gives the details of the period decomposition, the four censoring mechanisms (plus a fifth, deliberate censoring for AnimLOS, described in the Discussion), and the capping rule. The methodology incorporating truncation and censoring is valid at any scale (sample size), but its precision depends on scale (as it does for any statistical treatment). mLOS reports confidence intervals (see Section 2.1.7) and gaps in the risk set (Section 2.1.2) to caution against overextension.

Each stay record is converted into one row per period it participates in. An animal participates in a period if: it was taken in before the period ends; and either has not yet left or left on or after the period begins. Each participating animal-period pair yields an observation interval on the days-since-intake scale, together with an event indicator. The indicator is one only for a classified outcome recorded inside the period.

Four mechanisms produce a censored observation: censoring (at the period end) of an animal still in care at data extraction; censoring at a period boundary, where the outcome fell on or after the boundary and the same animal reappears, left-truncated, in the next period’s rows; capping at the restricted stay cap; and an unclassified exit, where the animal has a recorded outcome date but no classified outcome. All four are treated as noninformative. The first three are administrative and satisfy this by construction, because they are the consequence of the placement of boundaries (the study period, the data extraction date, and the cap) rather than a peculiarity of the stay itself. (An animal still in care lacks an outcome only because the data were extracted before it left.) The fourth is a design convention, discussed in Section 2.1.3 below.

To keep censoring noninformative, shelters should not choose period boundaries after the fact (from observations or analysis of census, intakes, and outcomes). But they can set boundaries at the dates of policy or operational changes, to obtain clearer contrast of LOS before and after each change. In this instance, censoring is noninformative, because the boundary date is set independently of the animals in care, and it models what would be expected in the absence of the operational change.

If the period boundary is set just prior to a new marketing program, stays in the pre-marketing period are censored at that boundary. The censoring means that, in the absence of the program, residents would continue with the same prospects that prevailed in the pre-marketing period. With marketing, their prospects improve, but that is not attributable to the pre-marketing period, and that makes censoring appropriate for contrasting the two periods. In the absence of milestone-driven period definition, a good default choice is pre-determined calendar intervals.

A configurable cap (named restricted_stay_cap in the settings) bounds the analysis horizon: any row extending past it is flagged as capped, shortened to the cap, and censored there. Capping makes all restricted-mean quantities well defined and prevents a few extreme stays from dominating means. The proportion of rows capped is reported. Because the cap sets the horizon for the restricted mean and for remaining length of stay, the tool’s primary point estimates, it is one of the most consequential settings.

For teaching purposes, a stand-alone script in mLOS (tools/histlos_by_period.R) computes HistLOS, the historical computation of LOS without truncation and censoring, by period. This script is not part of the standard mLOS run.

#### 2.1.2. Kaplan-Meier Estimator

Length of stay curves are estimated by the KM product-limit estimator [14], with the risk set at each event time computed from the counting-process intervals, so that truncation and censoring are respected. A restricted mean survival time (RMST) is computed from the KM survival curve. The restricted_stay_cap is the RMST horizon τ, standard in survival statistics [26].

With left-truncation it is possible for a gap to occur with no animals at risk, and the Kaplan-Meier estimator cannot recover from such a gap. The tool reports gaps in the pooled data and separately within each factor level. In the results workbook, gaps are highlighted in color.

When shelter staff process animals as they exit, they observe the LOS distribution given by the KM curve, which also describes the LOS prospects of animals at intake. But when shelter staff tour the kennels, what they see is not the LOS distribution but rather the distribution of in-care tenure: the days already spent in the shelter by each resident. Expressed in cumulative form like the KM curve, we call this the in-care tenure curve: for X days, the fraction of the residents with in-care days exceeding X.

The in-care tenure curve for the resident population is derived from the KM curve. At each value X, this is the integral of the KM curve above X, divided by its integral over the whole range (shown in the mLOS math methods documentation §5.7). That denominator is the restricted mean (RMST), so the result is a fraction rather than a count of days. The in-care tenure curve has a second interpretation. At tenure X, the tenure-curve fraction Y is the share of all animal-days delivered to animals after they have been in care for X days. At steady state, every resident with tenure above X accrues one animal-day per day, so the fraction of residents with tenure above X, is also the fraction of animal-days delivered to animals after X days in care.

The expected census at steady state for each tenure level by factor level, measured in animal counts, can be derived from the KM curves by multiplying each curve by average daily intakes. This curve allows comparisons of the number of animals in care (with a given tenure) across factor levels. A companion curve computed from KM shows conditional remaining LOS: the expected number of future days in care for a shelter resident, as a function of tenure.

#### 2.1.3. Aalen-Johansen Competing Risks

mLOS standardizes outcomes to three types: community live (L), such as adoption or return to owner; other live (T), such as transfer or foster; and non-live (N), such as death or loss of the animal. The mapping of shelter outcome codes to these types can be supplied in the settings file (or done upstream, e.g., in ShelterDataPrep [21]). These three types are a starting point that matches the distinction Shelter Animals Count makes between community and non-community flows [27]. A user-specified set of outcome types is planned as an extension. The three outcome types are treated as competing risks and estimated by the AJ estimator [19]. For an outcome type, the cumulative incidence function (CIF) is the probability of having left care by a given day with that specific cause (outcome). The AJ estimator accounts for the removal of animals by the competing causes. AJ is preferable to running KM separately for each cause (treating the other causes as censoring), because that would overestimate incidence [28]. The cumulative incidences sum exactly to the probability of leaving care, which equals one minus the KM survival probability. This is an additional reason to avoid the use of naïve per-cause KM [29].

From AJ, conditional outcome probabilities (probability of each outcome type conditional on tenure) are computed and plotted. For an animal still in care at the end of day X, this is the probability of leaving with outcome k by the horizon of the AJ analysis window. It is equal to the difference of CIF for outcome k between day X and the horizon, divided by the probability of LOS exceeding X days (the formula in the mLOS math methods documentation §7.4). These conditional curves can be read alongside those for remaining-LOS: they compute the expected outcome mix conditional on days in care, while remaining-LOS curves compute the time these outcomes will collectively need.

In an unclassified exit (Section 2.1.1 above), there is a recorded outcome date, establishing that the stay ended, but no classified outcome type. Such records are rare, because even the most basic shelter reporting relies on outcome types. The number of unclassified exits is reported in every run’s log, and the software warns when such stays reach 0.5% of the stays in the study window. (It is zero in the analysis window of the example; see Results section.) mLOS censors such stays at the exit date, as a deliberate convention. KM and AJ are fitted on the same rows, and their results are connected by an identity: the sum of the AJ CIFs equals one minus the KM survival function. The software verifies this in its tests. Counting the exit as an event in KM would require either breaking the identity or assigning a cause in AJ when the cause is not known. Creating a new cause for unknown outcome types would add complexity for the sake of extremely uncommon occurrences. Discarding the stay would be an option, but mLOS makes the design choice of censoring these stays, keeping the stay’s observed days at risk and withholding only the outcome. This censoring biases LOS slightly upward, in proportion to the number of such stays; in comparisons of outcomes in AJ, it effectively assigns such stays the outcome mix of later exits.

#### 2.1.4. Cox Proportional Hazards Regressions

The Cox model [15] is fitted for the hazard of all outcome types pooled, using dummy indicators for the main effects of each factor that has at least two observed levels, but no interaction terms. The partial likelihood is maximized over the counting-process rows, so the risk set at each event time contains exactly the rows whose interval covers that time, taking into account truncation and censoring. Reference levels can be selected by the user; by default, the reference is the oldest period and the most frequent level of the other two factors. Levels are ordered chronologically for periods and by character code (alphabetically, for plain upper-case labels) for intake types and animal groups.

Event times are whole days, so ties are widespread. The fit uses the Efron tie-breaking method [30] which targets the within-day rate ratio rather than the ratio of daily probabilities or daily odds.

The model is always fitted with clustering on the animal identifier that corrects the standard errors for the correlation among an animal’s multiple period rows or multiple stays. When the input-data CSV omits the identifier, one is generated per stay before the period decomposition, and that identifier gathers one stay’s period rows into a single cluster but cannot cluster repeat stays of the same animal.

In addition to the pooled form, a separate Cox regression is done for each qualifying factor, stratifying by all remaining factors. This is meaningful if at least two of the three factors are usable. It provides hazard ratios (assuming proportionality of hazards) for the levels of one factor, while giving every combination of the remaining factors its own separate baseline. This relaxes the proportional-hazards assumption for the remaining factors, leaving it in force only for the factor being fitted.

A separate runnable script (tools/cox_zph) applies the Grambsch–Therneau test of proportional hazards based on scaled Schoenfeld residuals [31,32,33] to the pooled Cox model. The script uses the cox.zph function of the survival package [18] with its default Kaplan–Meier transform of time and one test per model coefficient. It is not part of the primary mLOS run (which does not include diagnostics of hazard proportionality). Hazard ratios should be understood as averaged over tenure [34], not as constant multipliers. mLOS leads with cumulative measures, the KM curves and restricted means, and uses the Cox and Weibull regressions to answer one question: whether one factor’s ratios persist after adjustment for other factors.

#### 2.1.5. Weibull Regressions

When enabled in the settings, a Weibull regression is fitted on the available factors. It fits separate scale parameters for each factor level, but a single shared shape parameter which shows whether the discharge hazard falls or rises with tenure. Its coefficients exponentiate directly to LOS ratios. Using the shape parameter, implied hazard ratios are derived for comparisons to Cox, as a simple check of the Weibull description.

Weibull regression is also done on one qualifying factor at a time, allowing other factors to influence the shape as well as the scale (math methods §6.7). As with the per-factor Cox fits, this lightens the assumptions and allows an indirect check on the pooled Weibull. The pooled Cox and pooled Weibull regressions force one baseline shape onto every covariate pattern, so neither lets the discharge process vary between covariate patterns. Each per-factor Weibull shape variant keeps the identical scale formula, so the target factor’s LOS ratio stays directly comparable to the pooled Weibull’s, while the remaining factors are additionally placed on the shape parameter. A shape ratio near one says those levels share a discharge-hazard shape; a shape ratio far from one says their hazards evolve differently over the stay, a distinction the pooled models cannot see. Beyond the indicative value of confidence intervals, no diagnostics are provided for Weibull goodness-of-fit.

#### 2.1.6. Estimation of Census and Animal-Day Metrics

Total days of care provided and mean daily intakes are directly counted in the data. Days of care sum each stay’s days within the study window, counting both arrival and departure days. The mean intakes are needed in the computation of KM-derived statistics below. The mLOS math methods documentation contains an Appendix (Symbol Table) that defines the quantities used in this section and others.

There are three observable metrics that help summarize shelter population load per factor level: the mean daily census, the mean tenure of resident animals, and the accrued animal-days of the overnight population. The accrued animal-days are the care the resident population has already received: on an average day, each resident’s tenure summed across residents, equal to the census times the mean tenure per resident.

For each of the above three metrics, there is an observed value directly counted in the data as well as a fitted (model-based) counterpart computed from KM and its ancillary curves. The fitted census, reported as expected_census in the results JSON, is the prediction from Little’s law [35]: mean daily intakes multiplied by the KM restricted mean stay.

The mean tenure (per resident) is the area under each in-care tenure curve. The accrued animal-days are the product of census and mean tenure. Agreement between observed and model-based values is consistent with steady state (but is not proof of steady state); a gap suggests an unsettled transition.

There is an additional model-based metric, animal-days owed, which denotes the expected future animal-days of care the average in-care population will require. This is related to the curve of census profile (animal count by tenure level) and the remaining-stay curve. The element-wise product of these two, summed over tenures, is animal-days owed, i.e., the future care the standing population represents at each factor level.

Days owed minus animal-days accrued, both fitted, equals the census exactly. This is shown in the documentation (mLOS math methods §5.6 for this relationship and the derivation of days owed as a summation of expected remaining LOS). This relationship is part of the inspection paradox in renewal theory [36,37] where, for continuous time, the two quantities are equal. Under the end-of-day counting convention (math methods §2.7), a resident contributes, on the average, one more day forward than backward. Summed over the resident population, this makes days owed minus days accrued equal the census.

#### 2.1.7. Confidence Intervals

The core computations of confidence intervals are built into the R packages used and are covered in the mLOS math methods documentation (§5.2, §6.3, §7.3; see §8.4 for the list of CIs in the results workbook).

The confidence ribbon for the KM survival curve entails Wald intervals from the Greenwood variance on the log-survival scale [16,38] and the KM restricted mean follows the same approach but on the linear scale [39]. The fraction remaining at the restricted stay cap is read from the curve’s own bounds; it is blank if the curve reaches zero. The confidence intervals of percentiles (including the median) are derived by inverting the CI ribbon of the KM curve [16]; that is the standard approach for percentiles. The AJ ribbons and the AJ restricted mean days by state (days spent still in care, or already departed via each outcome, up to the cap horizon) are derived as Wald intervals from infinitesimal jackknife (delta method) approximations of the variance [18,40] on the log scale for the ribbons and linear scale for the means. KM intervals do not account for within-animal dependence when the same animal re-enters the shelter and may therefore be narrower than the true intervals. AJ intervals do cluster by animal id (if an id is provided). Since the all-cause AJ estimate (labeled “Any” in mLOS) is the complement of the KM curve, it can be used to assess the effect of animal re-entry on the confidence interval (plot ribbon and mean).

The intervals for mean daily intakes, outcomes, and incidence rates are computed from exact Poisson (Garwood method [41]). Proportions counted in the data rather than fitted (the fraction of rows reaching the cap and the outcome-mix shares) use exact binomial intervals [42].

For expected census, the confidence interval is computed from the daily intakes and the restricted mean survival time, with a delta method on a log scale. This interval is only indicative, as it treats the underlying two variables as independent. Moreover, daily intakes may vary more than a Poisson model allows, which would make the computed interval narrower than the true one.

For Cox hazard ratios, the computation involves Wald intervals on log hazard ratios (and the bounds are then exponentiated). The standard error is robust [43] and clustered on animal id (one id generated per stay if the actual id is not provided). The Weibull ratios also use Wald intervals on log ratios, but the standard error is derived from the Hessian (neither robust nor clustered).

Comparisons of pooled and stratified Cox hazard ratios (in the post-processing package, Section 2.2.5) use the errors of the ratios being compared (on a log scale), treating them as independent. Because the ratios are estimated from the same data and are positively correlated, the derived intervals are conservative (wider than necessary). The ratios of KM restricted means use the delta method on the log ratio [44], assuming that factor levels are independent.

We compute only those CIs that are available in the R packages and those that have a straightforward approximation. The normalized AJ restricted mean (mean days to outcome) and the conditional outcome probabilities do not receive confidence intervals because they involve ratios of correlated cumulative incidence values. Remaining-LOS curves, in-care tenure curves, and days-accrued and days-owed totals (past and future animal-days) likewise do not receive confidence intervals. These confidence interval computations may be added in the future.

### 2.2. Software Structure

Three pieces of software are involved, and they are connected only by files. ShelterDataPrep (version 0.4.0), written in Python (version 3.9.6), turns a raw shelter extract into the CSV that mLOS reads. The main mLOS software (version 0.3.0), written in R (version 4.4.2), performs every statistical computation and writes its results as plots, companion CSV files, a JSON file, and an Excel workbook. A third component, mlos_review (residing and versioned in mLOS but written in Python), reads the JSON, accesses the plots, and builds a slide deck. Each of the three can be replaced or improved without touching the others, as long as the connecting file formats are respected.

This article does not replace the documentation in the repositories. Rather, it gives an overview of the capabilities and methods, so that readers may ascertain the value of this software for shelter data analysis.

#### 2.2.1. Main Software System

mLOS is an R-based tool for analyzing animal shelter length-of-stay distributions and competing outcomes using statistical methods. mLOS works with shelter intake and outcome records standardized to three types of outcomes: community live, other live, and non-live. The records may also carry two categorical factors, intake type and animal group; the latter can be defined at runtime from other data fields. Consecutive analysis periods are defined by their starting and ending dates. The software partitions its analysis on each of these three categorical factors: calendar period, intake type, and animal group.

mLOS relies on techniques from survival statistics, but here survival means only that an animal is still in care, that is, has no outcome. The differentiation between outcome types, including live against non-live outcomes, is handled separately from length of stay, in the AJ competing risks analysis.

The analysis code is a flat set of R [45] (version 4.4.2) source files, one per stage. The script that orchestrates the full analysis is run from the command line “Rscript mlos_run_complete.R”, with optional arguments naming the settings file, the CSV data file, and the results directory. All three can also be set through environment variables, as can the names of the files written into the results directory. An unrecognized argument stops the run with a usage message.

A directory holds example input data and settings, with a provenance sidecar for each dataset. Generated output is held in separate directories, not tracked in the repository. Another directory holds the validation test suite: its fixtures, its committed expected outputs, and its runner.

The required R packages are survival [18] (version 3.8.9) and yaml [46] (version 2.3.10). jsonlite [47] (version 1.8.9) is additionally required to write the results JSON, and openxlsx [48] (version 4.2.8.1) to produce the Excel workbook; without either, the run still completes and the corresponding export is skipped with a warning. The flexsurv package [49] (version 2.3.2) is also needed when Weibull regression is enabled.

For users with no local R installation, the repository includes a Google Colab notebook that runs the same analysis in the browser. It installs the required packages and sets up the folders. The user must upload the R source files along with the data and settings files. A session takes several minutes to set up but can then carry out multiple runs.

#### 2.2.2. Shelter Data Used by the Software

Each row of the input CSV is one shelter stay, with mandatory columns for the intake date, the outcome date (blank meaning still in care), and the outcome type (blank, or one of three canonical outcomes: L for a community live outcome, T for another live outcome, N for a non-live outcome). The canonical codes are used internally, and a shelter’s CSV can either contain them directly or map onto them in the settings file. Optional columns are the intake type, the animal group, and an animal identifier. Any further columns are ignored by default, but they may be named in the settings file to construct an animal grouping or apply a one-off data filter inside the run. A shelter that houses multiple species should study them one species at a time. The animal-group feature is for distinctions within a species, such as by size or by gender.

The analysis is valid only if the data include all animal stays that overlap with the study window, not just those with outcomes in the study window. Truncation and censoring make statistically appropriate adjustments when the stay overlaps window boundaries.

LOS is calculated inclusively: an animal that arrives and leaves on the same calendar day has a length of stay of 1 day. There is no LOS of 0, as that would introduce complications in the underlying statistical methods which routinely require strictly positive event times so that the survival function equals 1.0 at time 0 [16]. In practice, this convention treats a kennel as occupied by an animal for the whole day even if the animal was only present for a portion of the day. Metrics (such as means and percentiles) derived here can be converted to the alternative convention of treating same-day arrival and departure as LOS of zero, by subtracting 1 from the LOS metric.

Integrity checks for duplicate stays and for physically impossible overlapping stays are applied to the complete file. The study-window trimming runs last, so a stay may influence the filtering of other stays even if it falls outside the study window and is destined to be dropped. This guarantees that altering the study window does not make animals enter or leave the study set for any reason other than their stay’s overlap with the study window.

Blank entries in the intake type and animal group columns are filled with a placeholder value, which can be dropped or mapped in the settings.

A settings file, written in YAML, defines the comparison periods and other options: how the outcome codes in the data map to the canonical L/T/N; data filtering in the run, mapping of values, and plotting options. For a standard run on a simple CSV file, only a few settings are required, the remainder assigned to default values when absent. An unrecognized setting name stops the run. Every setting is documented, with syntax and defaults, in the user guide’s “Settings reference” section.

#### 2.2.3. Software Output

A run produces a console log, a full set of Kaplan-Meier (KM) and Aalen-Johansen (AJ) plots as PNG files, additional plots computed from the primary KM and AJ, companion CSV files with the numerical values of the plots, and a JSON file with every numerical result other than the curve values. There is also a consolidated Excel workbook, which is a rendering of the JSON. Finally, the run writes a record of the filtering and mapping steps (both the built-in steps and the optional ones added by the user).

A quantity has a single name, in lower case with underscores, and it is the same name in the workbook, JSON, and CSV files. The JSON describes its own shapes and carries a manifest describing the plots and companion CSVs generated. Every curve and metric is produced for the whole sample and separately for each level of each available factor. The plot companion CSVs allow a user to plot results differently, including combining information from multiple factors and plot types.

The Excel workbook contains a descriptive cover sheet, the data-preparation ledger, the Cox regression, a whole-dataset sheet carrying the unified analysis, and one sheet for each factor that has two or more levels. It also provides Weibull sheets when the parametric regression is enabled. Where a quantity comes with a confidence interval, it is included in the JSON and workbook.

#### 2.2.4. Ancillary Package for Preparing Shelter Data

ShelterDataPrep [21] is a Python package upstream of mLOS. It reads a raw shelter extract together with a YAML settings file and writes the prepared CSV suitable for mLOS input, along with a processing statistics table recording what every filtering and mapping step removed or changed. ShelterDataPrep allows decoupling mLOS from shelter-specific formats and conventions.

While mLOS retains some filtering and mapping capabilities, the intent is for these to be used to explore alternate statistical analyses. mLOS writes its data-preparation statistics in the same column layout as the statistics table ShelterDataPrep writes, so the two files stack into one flow running from the raw extract to the rows the statistical models ran on.

With appropriate settings, ShelterDataPrep can convert disparate raw files into the same processed form. ShelterDataPrep can thus be used to enable studies of multiple shelters within the same run. Data from different shelters can be separately processed in ShelterDataPrep and converted into a designed shared format, usually including a field that tracks the source. These output files can then be manually stacked and presented as a single input file to mLOS.

#### 2.2.5. Experimental Software for Post-Processing Results

A slide deck builder resides in mLOS but is run separately. It is a Python package that reads the JSON and figures generated by R. It builds a pptx slide deck, together with a workbook of the tables it assembled. It does no sophisticated statistics of its own: it performs only simple transformations of the statistical results at hand and constructs simple plots, while embedding the existing KM and AJ plots in the slides. It attaches speaker notes to each slide and collects findings to present at the end, applying rules and thresholds.

The slide deck is not AI-generated. It is reproducibly constructed by Python code. When the data values shift, the deck changes in accordance with rules embedded in code. Decks have been tested for correctness, but not for utility and relevance in the field.

#### 2.2.6. Software Documentation

The repository contains complete documentation. After the standard introductory README files, the most important item is the mlos_user_guide, detailing every output file, plot, and setting. It includes a plain-language section for practitioners. A mlos_math_methods document with numbered sections (flagged by the symbol § in this article and in internal cross-referencing within the documentation) defines conventions, estimators, and derived metrics in sufficient detail to facilitate careful interpretation and reproducibility. Both documents are deposited with the software and versioned alongside it. They come in markdown form (authoritative and updated with every change on GitHub, even before release) and a docx form (derived only at release time).

Both mLOS and ShelterDataPrep tools are run via Command-Line Interfaces (CLI) which offer a “--help” option, take arguments at the command line or from environment variables, and protect against unrecognized arguments. mLOS also offers a documented Google Colab notebook as a path when R or its required packages are not installed locally.

#### 2.2.7. Software Testing

mLOS uses a five-layer test philosophy: hand-derived exact values; simulation-recovery; arithmetic invariants; golden files; and documentation-integrity checks.

A test suite, its fixtures, and its committed outputs reside in the tests directory, whose README documents every check. It also details the workflow for updating the committed outputs. Testing runs each fixture through the production analysis functions. Every analysis step is run on every fixture, including fixtures not built to exercise it, to find unhandled edge cases. One set of tests relies on small, hand-derived datasets that carry expected values (documented with their derivation), and these are compared exactly. This is a transparent way to test for correctness: an independent party can reproduce the expected values by hand (or in a simple spreadsheet) and compare them with both the documented fixtures and the program output.

Beyond the exact checks, the test suite includes six simulation fixtures, each a seeded sample from a fully specified model that can generate a new random sample on request. The simulations range from 1180 to 2909 stays (12,052 in total). Left truncation and right censoring arise from each fixture’s study window rather than being constructed: 5–10% of the stays in each fixture are left-truncated (829 in total), and 2–11% are right-censored (783 in total). Their expected values are the generating parameters. Each estimate is required to fall within about four standard errors of its generating value.

The simulated models include Weibull stays that differ by intake type; a true period effect that also applies to animals already in care; cause-specific rate changes that alter the competing-risk structure while the all-cause rate simply doubles; and a discharge shape set by the combination of intake type and animal group. One simulation is a shift in intake composition that produces Simpson’s paradox [50]: every dog type gets faster while the unified population slows down because the composition shifts toward the slower categories. Another simulation is unobserved heterogeneity that produces the declining pooled hazard [51]: a static mixture of large and small dogs produces falling outcome rates when size is ignored. The mLOS math methods documentation tabulates these fixtures (§10), their counts, and the known behavior each recovers, while the test suite requires that table to match the fixtures.

Another test layer checks the outputs rather than the estimates. Generated CSV files and the results JSON are compared byte for byte against committed copies, so that any change in output (intended or not) surfaces.

Arithmetic invariants are asserted directly on the results bundle, and quantities that the code computes by two independent routes are required to agree. Notably, the sum of the AJ cumulative incidence estimates must equal one minus the KM survival estimate. The output workbook is also rebuilt from the JSON alone and required to match the one rendered from live results. The slide deck builder carries its own tests.

Separate tests cross-check the illustrative numbers used in the user guide and math methods documentation, preventing drift between results and documentation.

There is continuous integration at the repository, with “.github/workflows/tests.yml” running the R suite and the Python suite on every repository push: the R suite under two locales (to confirm that the pinned sorting of category labels holds) and the Python suite across Python 3.9 and 3.13 with two pandas major releases (2 and 3).

The software uses standard reproducibility practices. Console log files stamp source and output SHA-256 [52]. Prior to generating any results files, existing prior results are auto-archived to avoid accidental overwriting and facilitate comparisons. There is version control and DOI deposit of each release [53] supported by deposits of the input data and versioned results [54].

## 3. Results

To illustrate the methodology, this section uses example plots and tabular results from the OC2 run deposited in the main results [55] and the slide deck [56]. The underlying tables and figures are generated by an mLOS run (plus slide deck build) on the deposited input, with no post-processing, and their source files are named. Panels within a figure share an axis and are meant to be read together.

The example involves dogs in Orange County Animal Care. It uses three periods, bounded by 2019-01-01, 2020-04-01, 2021-07-01, and 2022-10-01 (stated here in the YYYY-MM-DD format required by the mLOS settings file) and labeled Pre-C, Early-C, and Late-C (referring to the COVID-19 pandemic). This window contains 15,788 stays of 13,346 dogs: these are the stays that overlap with the study window and thus contribute to the example. (The OC2 deposited dataset [57] is wider, containing 34,718 stays of 28,230 dogs in care between 1 June 2018, and 2 October 2025: This enables studies for other calendar periods to be done from the same dataset.) Examples with an early version of the software [17] used the same periods but different data filters and factor level aggregation; that older dataset is still in the repository (labelled OC1 [57]). The dataset used here (labelled OC2) is the one given in the Data Availability Statement.

We examined the data set for the unclassified exits discussed in Section 2.1.1 and Section 2.1.3. In the raw Orange County data [58] (the starting point for ShelterDataPrep [21]) there are four dog stays with non-blank outcome date but blank outcome type. Of these, three entered the shelter already dead (a common occurrence, including disposal of animal remains or a head test for rabies) and were removed by ShelterDataPrep. Only one carries over into the OC2 processed data [57] (output of ShelterDataPrep and input to mLOS). This stay is removed in the last stages of mLOS filtering, because it occurs in 2025, outside the study window.

In this example, the higher LOS and lower hazard ratios for the Late-C period are associated with restrictions in visitor access to kennels [5,10,11,17,59] that remained in effect after other shelters re-opened; that is the insight the shelter could have gained from mLOS, had it been available in 2022. That is the reason the corresponding example of an earlier software version uses plots by calendar period [17]. Here, the example illustrates mLOS more broadly, including the effects of intake type and animal group (the latter chosen to reflect dog size).

### 3.1. Kaplan-Meier Length of Stay

Figure 1A shows the KM curves by animal group, which in the OC2 example is dog size. Though plots end on day 58, the horizon of the curves is the restricted_stay_cap set to 365, and the full curves exist in the results as CSV files. The restricted mean survival time is the area under the fitted curve up to that horizon [39] and it is included in the plot’s companion CSV. Figure 1B shows the in-care tenure curve for the resident population by animal group. The difference between the panels of Figure 1 is a consequence of the heavy tail of the LOS distribution: the population standing in the kennels (Figure 1B) skews long relative to the arriving cohort (Figure 1A). Figure 2 shows a pair of panels like those of Figure 1 but with curves partitioned by intake type.

The median and the 90th percentile (which may or may not be reached) are included in the results along with the restricted mean in the repository’s workbook [55]. Large dogs have 3.75 times the restricted mean stay of medium dogs (30.6 against 8.2 days) but 6.2 times their mean resident tenure (70.1 against 11.3 days). _UNKNOWN_ is the placeholder the tool substitutes where the size field is blank, left in the data so that every stay is accounted for. The MED and SMALL curves nearly coincide, their restricted means being 8.16 and 8.64 days: either medium and small dogs genuinely share a length-of-stay distribution, or there is a data-quality issue (size assignment at intake) requiring further investigation.

Table 1 gives LOS metrics by period from the workbook [55], with confidence intervals. A single central measure conceals movement in the long-stay tail: the median is a small integer and hardly moves across periods. From the Pre-C period to Late-C, the median is unchanged, but the restricted mean rises by half, and the 90th percentile roughly doubles.

The historical computation (HistLOS) gives a different picture of the same data. Its restricted means are 14.0, 15.9, and 19.9 days for Pre-C, Early-C, and Late-C, against 14.3, 14.5, and 21.9 days for ExitLOS in Table 1. HistLOS shows stays lengthening by 14% from Pre-C to Early-C; ExitLOS shows only a 2% change, its restricted mean ratio being 1.02 (95% CI 0.93–1.11). HistLOS understates the Late-C increase over Pre-C (43% against the ExitLOS 53% from the table).

The last block is the diagnostic for the restricted stay cap; well below 0.01 throughout, so the cap (365 days) is not shaping these estimates. This example underwent screening as shown in the settings files in the repositories for both the preparation program ShelterDataPrep [21] and for mLOS. The sensitivity of the restricted mean LOS to the cap can be computed from the KM estimate of the fraction still in care at the cap.

The overall restricted mean is 16.731 days and the KM estimate for the probability of reaching the cap is 0.00339. Thus, the mean changes by 0.00339 (0.02%) per day of movement of the cap. Naturally, the sensitivity increases as the cap draws in and decreases as the cap is pushed out, but changes happen only at event times (i.e., on days with a recorded outcome). The restricted mean itself changes over intervals with or without events, and becomes constant only when the KM curve reaches 0. Moving the cap from 365 to 345 or 385 changes the overall mean by 0.07 or 0.06 days respectively. By stratum (factor level), the largest change is 0.20 days (OWNER intake type). All changes are within the confidence intervals of their baseline restricted means. At a cap of 345, the highest KM fraction in care is 0.0103 for LARGE dogs, also within the margin of error of the fraction at the 365-day cap (0.0066–0.0115).

At tenure X, the in-care tenure fraction Y equals the share of all animal-days delivered to animals after they have been in care for X days. Read Figure 1B at X = 30: LARGE is 0.56, so more than half the care given to large dogs goes to animals after they have been resident for a month, against 0.10 for MED and SMALL. At X = 7 these read 0.81 for LARGE against 0.40 and 0.41. At the right edge of the plotted range, X = 58, LARGE still has 0.40 of its animal-days beyond, against 0.03 for the other two.

The expected census for each tenure value by factor level, measured in animal counts, allows comparison of the number of animals in care (with a given tenure) across levels. Figure 3A allows this comparison by animal group, measured in animals rather than probabilities, so that the area under a curve is that group’s expected census. Figure 3B shows conditional remaining LOS: the expected number of future days in care for a shelter resident, as a function of tenure. The pointwise product of the curves in Figure 3A,B, summed over tenures, is the animal-days owed, i.e., the future care the average resident population represents. As noted earlier, _UNKNOWN_ is a placeholder for an empty field, and MED aligning with SMALL may be rooted in the label assignment at intake.

### 3.2. Census and Animal-Day Metrics

Table 2 gives the totals these curves produce, effectively providing workload metrics by animal group, counted and fitted. Agreement between counted values (read from the records) and fitted values (steady-state predictions from the LOS curve and the observed intake rate) is consistent with a population near steady state. The mean tenure (per resident) is the area under each in-care tenure curve (these are the curves in Figure 1B).

In the first block, large dogs are 38.4 percent of intakes and 69.7 percent of the days of care given, so they consume care at nearly twice the rate their share of arrivals would suggest. Medium and small dogs run the other way, together 50.1 percent of intakes and 25.6 percent of the days. Measured in animal-days accrued, or future animal-days expected, the comparison tilts further: 92.7 percent of the owed care is attributable to resident large dogs. This is the length-bias effect of Figure 1, in animals and days rather than probabilities.

### 3.3. Cumulative Incidence by Outcome Type

Three cumulative incidences are shown in Figure 4, by animal group (dog size): Figure 4A is for community live outcome L; Figure 4B for other live outcome T; and Figure 4C for non-live outcome N. Each curve is estimated with the competing outcomes properly accounted for rather than treated as censoring. The sum of the three outcome curves, added to the KM curve (which measures fraction remaining in care), adds up to 1. As noted earlier, _UNKNOWN_ is a placeholder and MED aligning with SMALL may be a label assignment issue.

All-cause AJ are also generated (their plots are in the results repository [55] but not presented here). Their estimates equal 1 minus the corresponding KM curves. Because, as described in Methods, AJ clusters standard errors by animal id, we compare the confidence intervals for the AJ mean time in care to those of the KM restricted mean. Across all strata, the clustered CI ranges from 3.3% narrower (animal size _UNKNOWN_) to 4.2% wider (intake type RET, which is the category likely to have many animals with multiple stays). No limit moves by more than 0.13 days. CIs for medians are identical in every stratum. CIs for P90 only move by one day for 3 of the 24 stratum limits, and that is 10% or less of the width of the CI. These differences are, in practice, negligible.

### 3.4. Regressions

For Cox and Weibull regressions, the worked example uses the Pre-C period, STRAY intake type, and MED animal size as the reference levels. A diagnostic script for the proportionality of hazards (see tools/cox_zph in Methods) applied to the pooled Cox model rejects proportionality for most coefficients: 7 of 9 at *p* < 0.002; global χ^2^ = 605 on 9 degrees of freedom, *p* < 0.001. The pooled hazard ratio is therefore a summary averaged over tenure [34], not a constant multiplier.

The KM curves, restricted means, and Cox and Weibull regressions agree that Late-C stays were longer. Figure 5 compares different computations of hazard ratios and LOS ratios. The comparisons do not formally establish that the underlying modelling assumptions hold but act as informal sensitivity checks. Qualitatively, hazard ratios and LOS ratios run in opposite directions: a period whose animals leave faster has a hazard ratio above one and an LOS ratio below one.

Figure 5A shows hazard ratios by period: from the pooled Cox regression, from the per-factor stratified Cox fit, and inferred from the pooled Weibull regression. Comparison of the two Cox ratios to each other is a check on the sensitivity of each factor’s hazard ratio to the proportional-hazards assumption for the other factors. Agreement between Cox and Weibull suggests the Weibull form is also a reasonable approximation for estimating ratios in this example.

Figure 5B shows LOS ratios: from the pooled Weibull regression, from the Weibull with the other factors’ shapes freed, and the unadjusted ratio of restricted mean LOS computed from the KM survival curves. Agreement between Weibull estimates suggests that the shared shape parameter performs adequately for the purpose of estimating ratios here. The deviation of KM suggests that the LOS changes were affected by changes in the mix of intakes and animal groups. However, all regressions (as well as the KM) show a substantial increase in LOS (reduction in hazard rate) in Late-C.

The Cox and Weibull results are not intended to supplant the KM curves and means. Rather, the regressions demonstrate that the Late-C increase in the KM comparisons is not an artifact of changes in the intake type mix or the animal group mix. More generally, they can show whether an apparent effect of one factor reflects changes in the mix of levels of other factors.

Without additional investigation, the analysis produced by mLOS should be taken as descriptive rather than evaluative or prescriptive. The causal interpretation of the Late-C increase (restricted kennel access) is supported by additional research that showed that viewable animals were adopted at much higher rates [59], and that during viewing periods slow-track animals had adoption rates 1.5 times as high as during non-viewing periods [5].

## 4. Discussion

mLOS is a tool that helps animal shelters answer questions about length of stay (LOS), and how it changes over time. The tool usually provides LOS distributions, rather than just averages, and connects LOS to metrics about census, days in care, and flow of animals. It is intended for the study of animal shelter metrics: changes over time, comparisons across facilities, and operationally useful partitions of shelter animals. The tool produces plots, tables, and metrics that help the shelter assess its operation and identify priority areas for improvement. The tool can be used iteratively: a shelter can instantly refine and refocus the analysis through additional runs using data filtering and mapping.

In the OC2 example, large dogs dominate the population in care, especially at long tenures, even though the intake is more balanced across sizes. From the Pre-C period to Early-C the mean LOS held constant as intakes fell; but then in Late-C with intakes only modestly higher than Early-C (and still much lower than Pre-C) the mean LOS rose, and with it the census. The effect is stronger at the tail of the distribution (the longest-staying fraction) and can be visualized over the range of days in care. In regressions that include dog size and intake type, a statistically significant period effect remains.

The illustration included only a small sample of the comprehensive results in the repository [55]. A shelter can replace the OC2 data with its own (cleaning and preparing it with ShelterDataPrep [21], if that is helpful). After choosing the periods and animal groups of interest, it can produce a complete analysis for its own data. With two or more levels in each factor (periods, intake types, and animal groups), the structure of the generated results matches OC2 (i.e., the results contain the same plots and tables). From these, the deck builder module builds a slide deck analogous to the OC2 deck [56]. The slide deck relies on threshold rules and its selection of levels to tabulate under each figure may differ, but it also provides a separate workbook with the complete table.

Generalizing beyond the example, below are the types of questions that the software can help shelters answer (condensed from a longer list in the mLOS user guide).

LOS over time and across groups: Which categories of animals stay longer or shorter? Did length of stay change after we shifted a policy? By how much? Could the difference be just noise? Even if the typical stay is holding steady, is the long-stay tail growing or shrinking?

How an animal’s prospects change during its stay: If an animal has already been here two weeks, how many more days should we expect it to stay? Does the likely outcome change the longer an animal stays? Among the animals still with us at two weeks, what outcome mix should we expect from here on?

Outcomes and how they compete: Do community outcomes (adoption, return-to-owner) occur faster or slower than non-community (transfers)? What fraction of animals reaches a live outcome within their first week, or their first month? Are non-live outcomes concentrated early in the stay or late? Has the timing changed?

The population in care and animal-days load: How is the mix of animals in care different from the mix seen at intake? How do the animals in our kennels skew toward long stays compared with the animals we take in? Does our average daily census match what our intake rate and stay lengths imply? How much future care (animal-days of work owed) does our average resident population entail? Which group would free up the most animal-days if we could speed up its outcomes?

Filtering and partitioning: Do LOS patterns differ by intake type (stray, owner-surrender, or other)? Do they differ by animal size, gender, shelter facility, or another grouping available in our data?

Emphasis on full curves: For each day from intake on, what fraction of animals are still in care, how many days of care remain. Do animals in some category fall behind in the first two weeks but then catch up or overtake? Does the expected outcome shift with days in care?

Policy changes (what shelters have control over) are a motivation for shelters to look at metrics. However, a metric change is something to investigate and does not, in itself, prove that there was an adverse or beneficial change, in policies or in the community being served. Likewise, a comparison among shelters describes how they differ but cannot, on its own, judge shelter decisions. Shelter practitioners are familiar with some metrics already (typically the Live Release Rate) and know that a change in an aggregate metric does not reveal why or how the change is happening. All users of this software should be vigilant about handling data conventions, screening for errors, and presenting results in a manner mindful of shelter circumstances. mLOS does not encourage overinterpretation of metrics, but rather removes the barriers to their computation and enables their refinement by factor and level.

### Limitations and Extensions

The rest of the discussion reviews the limits of the current system and potential future adjustments and extensions. The system’s evolution depends on an assessment of user needs and interests, which could not be ascertained prior to software release and this article.

There is a tradeoff between guardrails and flexibility. The code takes only limited steps to enforce data integrity and may halt the run. Some issues can be addressed in the settings file. One example is how row-level data errors are handled. By default, the run stops immediately with an error message describing the problem. Setting discard_bad_rows to true instead drops the bad rows, prints a count, and continues the data processing. A similar option is offered by discard_overlapping_rows for stays of one animal that overlap in time, which is physically impossible: the run either stops or drops the shorter stay. Another example is unknown intake type. By default, the code will convert it to its own label, _UNKNOWN_. But, in the settings file, a filter can drop the rows with unknown intake type; or a map can merge _UNKNOWN_ into another existing intake type. Users are advised to check console logs and descriptive statistics to avoid unintended behavior.

The restricted stay cap imposes constraints on modeling the longest stays and distinguishing true outliers from merely uncommon long stays. KM does not model behavior past the cap, but its restricted mean effectively assumes exit at the cap (while AJ restricted time-to-outcome excludes animals that reach the cap). The cap is the horizon τ needed in restricted-mean methods in survival statistics [26]. If the longest stays are censored (animals still in care at the end of the period), the mean is not identifiable without a horizon. Past the last observation, the restricted mean is an extrapolation. If the longest stay in the stratum being analyzed (which may be the whole dataset or one factor level) is a classified outcome, the cap becomes effectively optional. A shelter confident in its data could set the cap to a high value (e.g., 9999). Any cap value beyond the longest stay has no effect, and the longest stay becomes the effective horizon. This exposes the shelter to the risk from data quality and outliers. A comparison among strata may hinge on one outlier.

Care should be exercised when animals do reach the cap and metrics sensitive to the cap are used. In practice, the considerations are those typically faced when deciding to cap an outlier. The less a dataset is understood and cleaned, the more caution is warranted. Note that this is not about discarding outliers, as capped animal stays participate in the computation up to the cap. Long stays are not discarded (except in the rare case where the entire stay lies beyond the cap). The user guide’s section “Choosing your restricted_stay_cap” discusses the tradeoff and the diagnostic for whether the cap is shaping a result. Additional runs with different caps are the simplest way to check the sensitivity of a metric of interest.

The three outcome types give practitioners a way to examine the interplay between the LOS information in KM curves and outcome rate information in AJ curves. Limiting the initial set to three categories avoids proliferation of plots and tables (including entries in the results workbook) and encourages practitioners to focus on length of stay. A planned extension is to make AJ outcome types user-defined. Animals still in care when the restricted stay cap is reached are censored there, so they appear as a wedge of unresolved probability at the top of the conditional outcome charts. Treating that remnant as an outcome type in its own right is a candidate extension [17]. It would resolve the missing wedge, and it would put the animals whose stays the cap does not resolve on the same footing as the three recorded outcome types.

Each stay in the input file is treated as separate. If an animal has multiple intakes, each is a separate record. The animal_id column is used for clustering standard errors in AJ estimates and Cox regressions. KM treats stays as independent, so its intervals may be slightly too narrow when animals return; its complement, the all-cause AJ CIF, carries clustered intervals for comparison. The animal_id is also used for detecting duplicate or overlapping stays for the same animal. But mLOS does not attempt to link successive stays analytically. Such linkage is possible in future versions and may allow computation of the LOS of an animal returned within a few days of adoption: the post-return stay can be left-truncated and include the days of the first stay.

It is left to users to deploy factors or levels only to the extent that confidence intervals remain satisfactory. With very few events per level, confidence intervals are wide and tests underpowered. However, days-accrued and days-owed totals (past and future animal-days), the mean days to outcome in AJ, conditional outcome probabilities, remaining-LOS, and in-care tenure curves currently lack confidence intervals. These may be added in future versions.

When referring to animals in care and their metrics, mLOS currently provides only model-based steady states or averages over a period. Steady state here does not require strictly constant intake rates. Rather, the operative assumption is that the calendar period being analyzed is not in the midst of a transition at a time scale close to the duration of the period. How long a transition takes to settle depends on the LOS distribution (including its tail). If a change is underway at time scales much longer than the period being studied, then the period itself will still approximate steady state. Notable discrepancies occur primarily when a change is underway, or occurred just before the period, and the period itself is still processing the transition. The fitted metrics should simply be understood as the predictions of the KM estimates, treating the stays in the period as a sample from a stationary process; the counted metrics carry no such assumption, and a gap between the two suggests a period still in transition. More general consideration of shelter dynamics is outside the scope of the software.

mLOS reports metrics for the average resident population (including by stratum) but does not analyze a shelter’s current population or any other point-in-time snapshot. Analysis of current residents (supplied separately, or taken as of the last date of the study period) is not difficult in principle and may be added in the future.

Each of the two alternatives used for simultaneous modeling of factors makes an assumption. Cox regression assumes that hazard ratios are constant over the entire LOS range. It does not produce an LOS ratio. Violations can occur when, for example, long-stay animals have a fundamentally different discharge profile. The per-factor stratified Cox fits are a partial screen on this: they re-estimate each factor’s hazard ratios without assuming proportional hazards for the other two. A large gap between one of these and the pooled Cox is a sign the assumption is straining for the remaining factors. Each factor’s sheet in the results workbook carries this fit’s hazard ratios beside the pooled ones; its global tests and concordance are in results.json only. Weibull assumes a specific form of the LOS distribution but (appropriately formulated) yields LOS ratios which are more meaningful to practitioners.

The hazard ratios in the Cox regressions should be understood as averages over tenure; their reading should be guided by the cumulative measures (the KM curves and restricted means). Visual inspection of KM curves for crossing behavior is an initial check for gross violations of proportionality. A stand-alone script (tools/cox_zph) available in mLOS runs the Grambsch–Therneau test of proportional hazards [31,32,33]. Further formal and visual diagnostics for the proportional-hazards assumption in Cox regressions are a possible future addition, but they may or may not be integrated into the primary run. Other significance tests between the pooled and per-factor Cox models (or the corresponding Weibull models) may also be added if these regressions are valuable to practitioners. Regardless of proportionality, the regressions aid in assessing whether an effect observed in one factor may be largely explained by changes in other factors. A possible refinement is hazard ratios within tenure ranges, but these condition on animals still in care and are subject to selection [60]: if a shelter moves its more adoptable animals out sooner, it is left with harder-to-place residents, and can show lower hazards at later tenures while being better off cumulatively. Diagnostics that localize non-proportionality invite exactly that misreading [61] and the cost of explaining it would outweigh the benefit for practitioners. Diagnostics for goodness-of-fit for Weibull regressions are also a possibility, but with a lower priority than Cox; Weibull regressions currently consume most of the execution time and are less practical for highly interactive runs.

The description of outcomes in mLOS by default corresponds to ExitLOS [10,11,17], a length of stay measured up to the animal’s exit from the organization that took it in, whatever form that exit takes. It contrasts with HistLOS, the historical method which restricts the calculation to the animals whose outcomes fall inside the period. An alternative view, AnimLOS [10,17], may be useful in comparing shelters regionally, or in assessing an animal’s total experienced LOS across organizations. This can be implemented in the mLOS settings file, by moving the raw codes that denote transfer to another organization to outcome_type_censored. This recodes them as censoring instead, since such a stay continues elsewhere but is not captured in the data. With this setting, mLOS effectively computes AnimLOS. (The recoded stays are censored like the unclassified exits of Section 2.1.3, here by intent, and the two counts are reported separately. AnimLOS does not alter the run’s warning about unclassified exits.) Given the assumptions involved, AnimLOS should not be treated as a prediction of total length of stay, but rather as an indicative first-order correction for previously unaccounted post-transfer stay.

The competing-risks analog to Cox regression is the Fine-Gray regression [62] that models subdistribution hazards, linking predictors to outcome-specific cumulative incidences. This regression may be added in a future version. Outcome-specific curve shapes are distorted early in the stay by mechanistic constraints such as a hold period. The all-cause outcome curve is smoother, the irregularities largely offset, so that the underlying distortions have little impact on the Cox regression. For AJ CIF curves the shape simply carries through to the plot. But in a regression such distortions may require more careful treatment.

The experimental slide deck builder has the most limitations, detailed in its documentation. It does not rank factors against each other, trade slides off against a budget, or aim for a pre-set detail level. Further development can sharpen its selection of factors and levels highlighted; its juxtaposition of related plots; and its settings that tailor the presentation to a particular audience type. The slide deck’s companion workbook, which currently holds the tables the deck build made, can be expanded into a stand-alone report.

The Google Colab notebook is a stand-in for a hosted interface rather than an end state. It removes the need for a local R installation, which may be a major barrier for shelter staff, but it loads packages each time and requires the user to upload source files. Hosted versions or incorporation into shelter database software are the preferable solutions, to remove the need for local installation of languages and modules.

The software is a reference implementation that can grow both in capabilities and availability. In addition to capabilities envisioned above, feedback from users may correct errors and identify other needs that the software can meet via extensions. A hosted version or database integration may spur applications and inform the directions for future development.

## 5. Conclusions

The distinctive contribution of the tools presented here is the integration of statistical methods around shelter-specific analysis needs, going beyond the simple accounting view of animal data currently implicit in shelter databases. The rigorous analysis of LOS by calendar period is the centerpiece of the tool, because it enables comparison of one period to another for the same shelter, as well as comparison of a shelter to a peer for a fixed calendar period.

The mLOS tool presented here characterizes how stay lengths vary across groups and shift over time; distinguishes between movement in the median and changes in the long-stay tail; and estimates how an animal’s prospects change conditional on time already served. It compares competing outcomes in both frequency and timing. It relates intake composition to the composition of animals in care, and it quantifies the animal-days of care the average resident population still represents. Analyses can be stratified by intake type and animal grouping chosen by the shelter. Throughout, mLOS reports complete distributions alongside point summaries. Confidence intervals are computed for many (but not all) metrics.

Shelters that apply mLOS and its associated tools to their data can track their own metrics, compare them to those they read in studies or reported by their peers, and gain a quantitative understanding of segments of their animal population and the days of care each segment requires. Metrics can then motivate operational interventions.

## Figures and Tables

**Figure 1:**
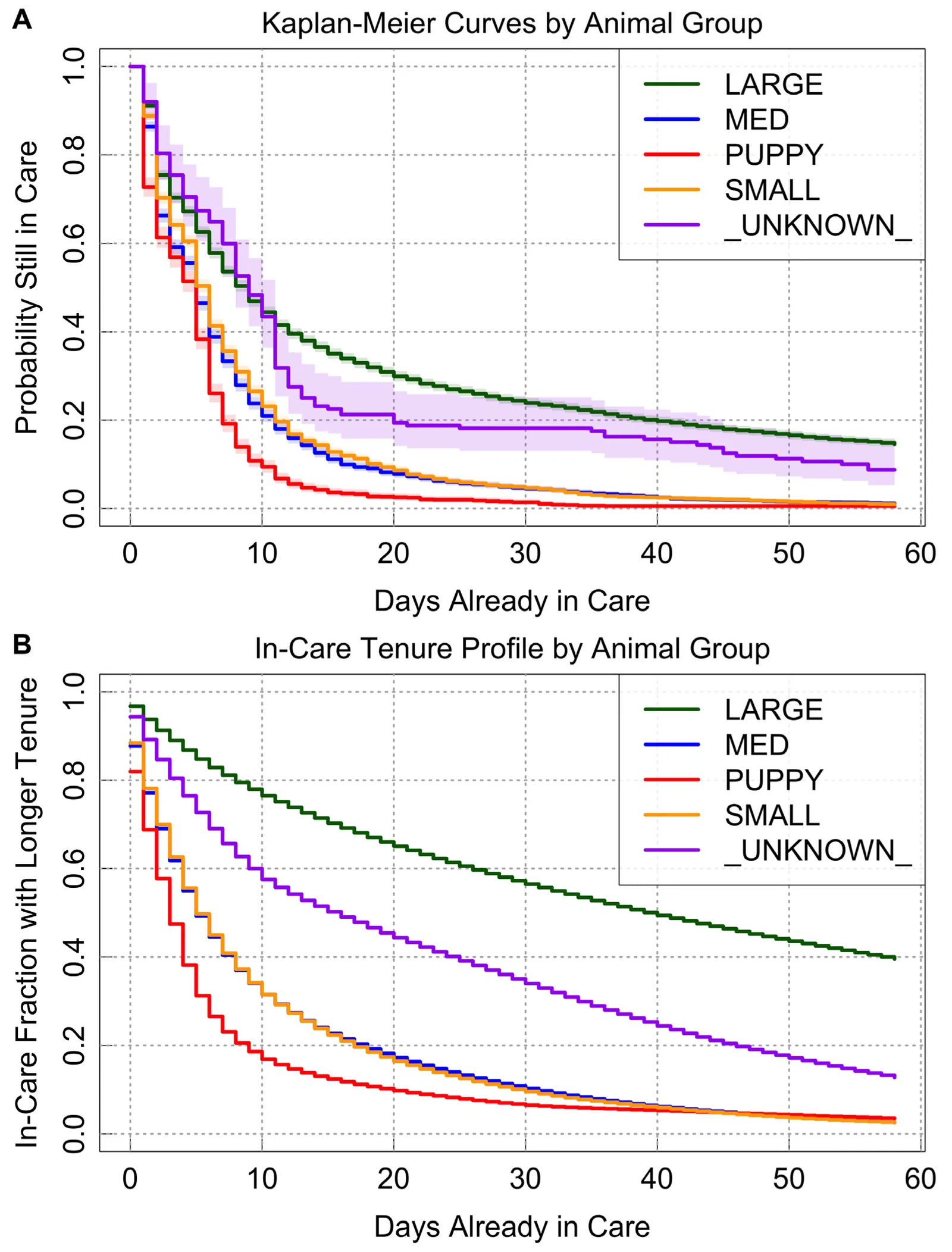
**Length of stay and resident tenure by dog size.** (**A**) The estimated probability that a dog taken in is still in care, by days since intake. (**B**) The fraction of the in-care population whose tenure already exceeds that many days. The two panels describe different populations on a common axis: the arriving cohort and the population standing in the kennels. Source files [55]: km_survival_by_animal_group and km_in_care_tenure_by_animal_group.

**Figure 2:**
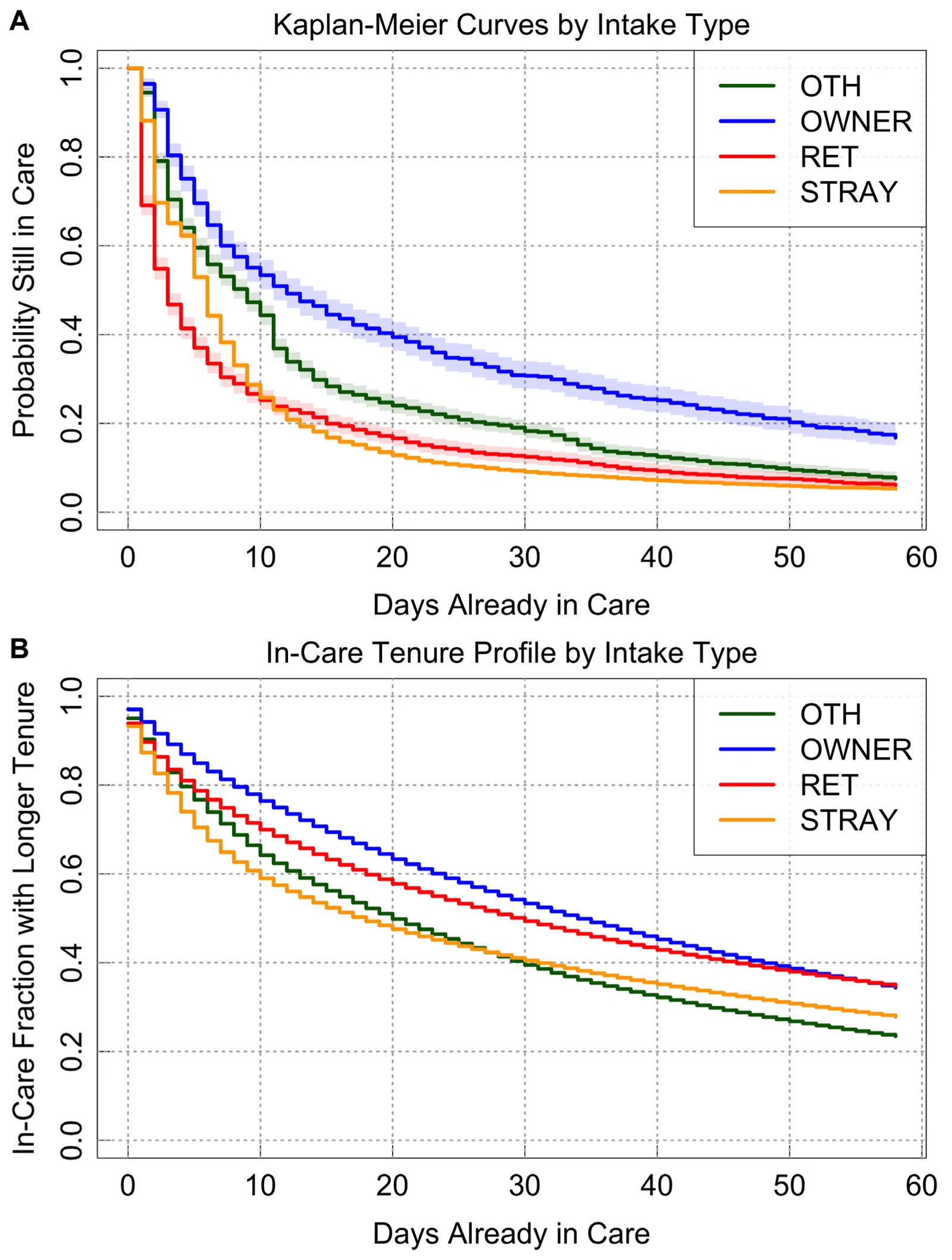
**Length of stay and resident tenure by intake type.** The same two quantities as Figure 1, on the shelter’s other categorical field. (**A**) The probability of still being in care by days since intake. (**B**) The tenure profile of the in-care population. Source files [55]: km_survival_by_intake_type and km_in_care_tenure_by_intake_type.

**Figure 3:**
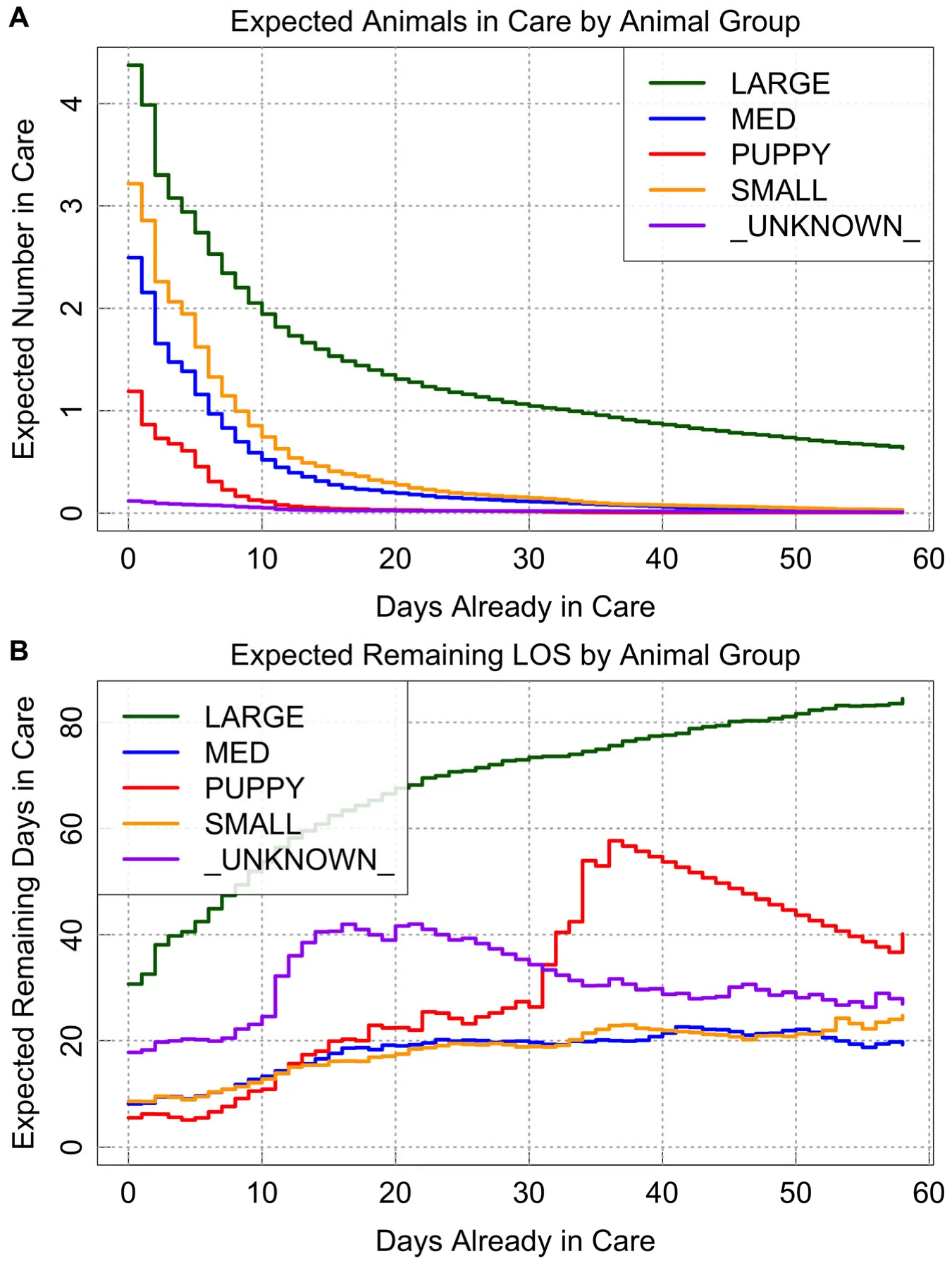
**The standing population and the care it still owes, by dog size.** (**A**) The expected number of dogs in care at each tenure, in animals rather than probabilities. (**B**) The expected further days in care for a dog that has already been there that long. Source files [55]: km_census_by_tenure_by_animal_group and km_remaining_los_by_animal_group.

**Figure 4:**
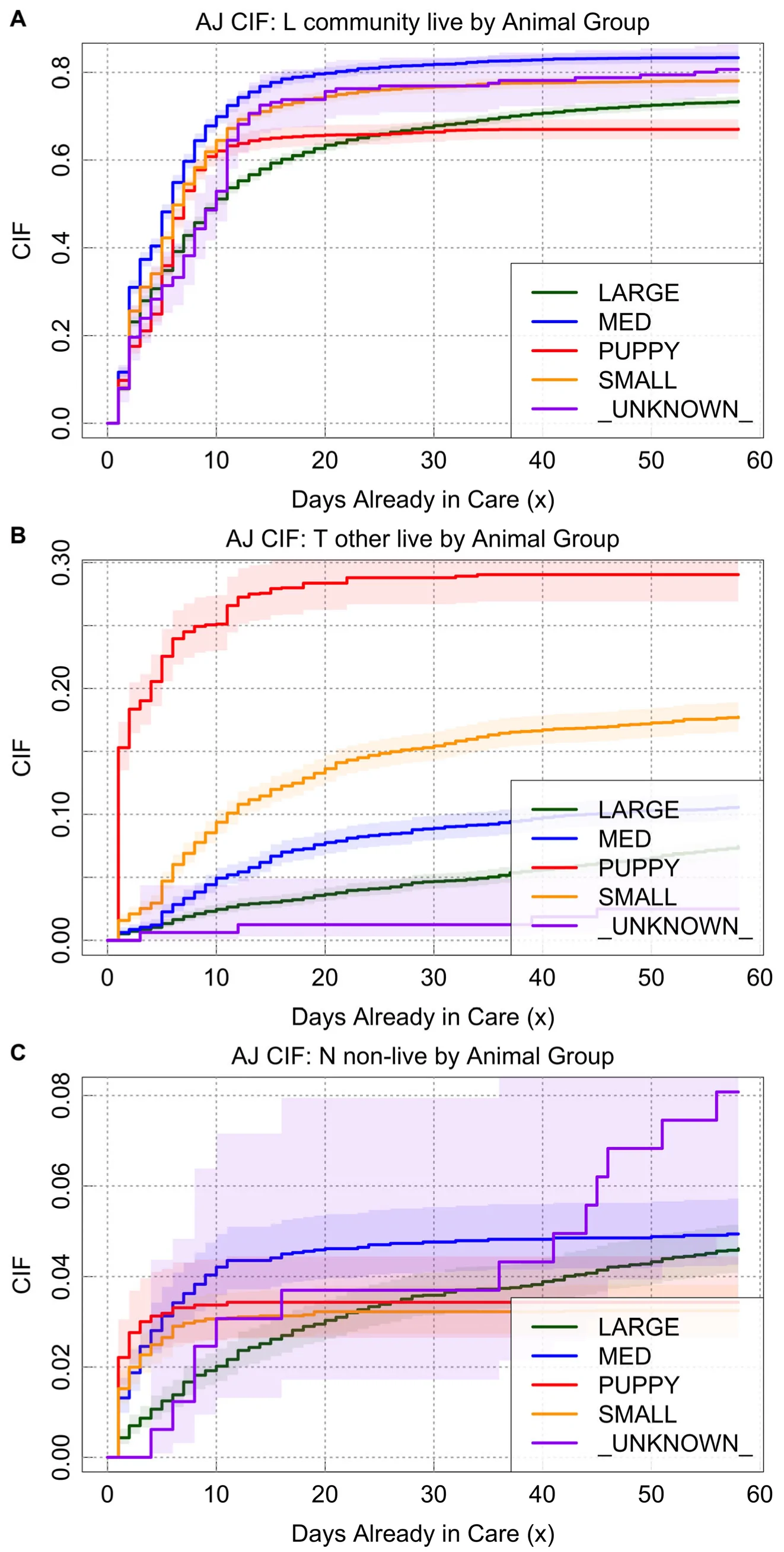
**Cumulative incidence of each outcome type by dog size.** (**A**) Community live outcome, encompassing adoption or return to owner. (**B**) Other live outcome, such as transfer or foster. (**C**) Non-live outcome, encompassing death or loss of the animal. Each curve is the probability that a dog has left care by that day for that specific reason. Source files [55]: aj_cif_by_animal_group_outcome_L, aj_cif_by_animal_group_outcome_T and aj_cif_by_animal_group_outcome_N.

**Figure 5:**
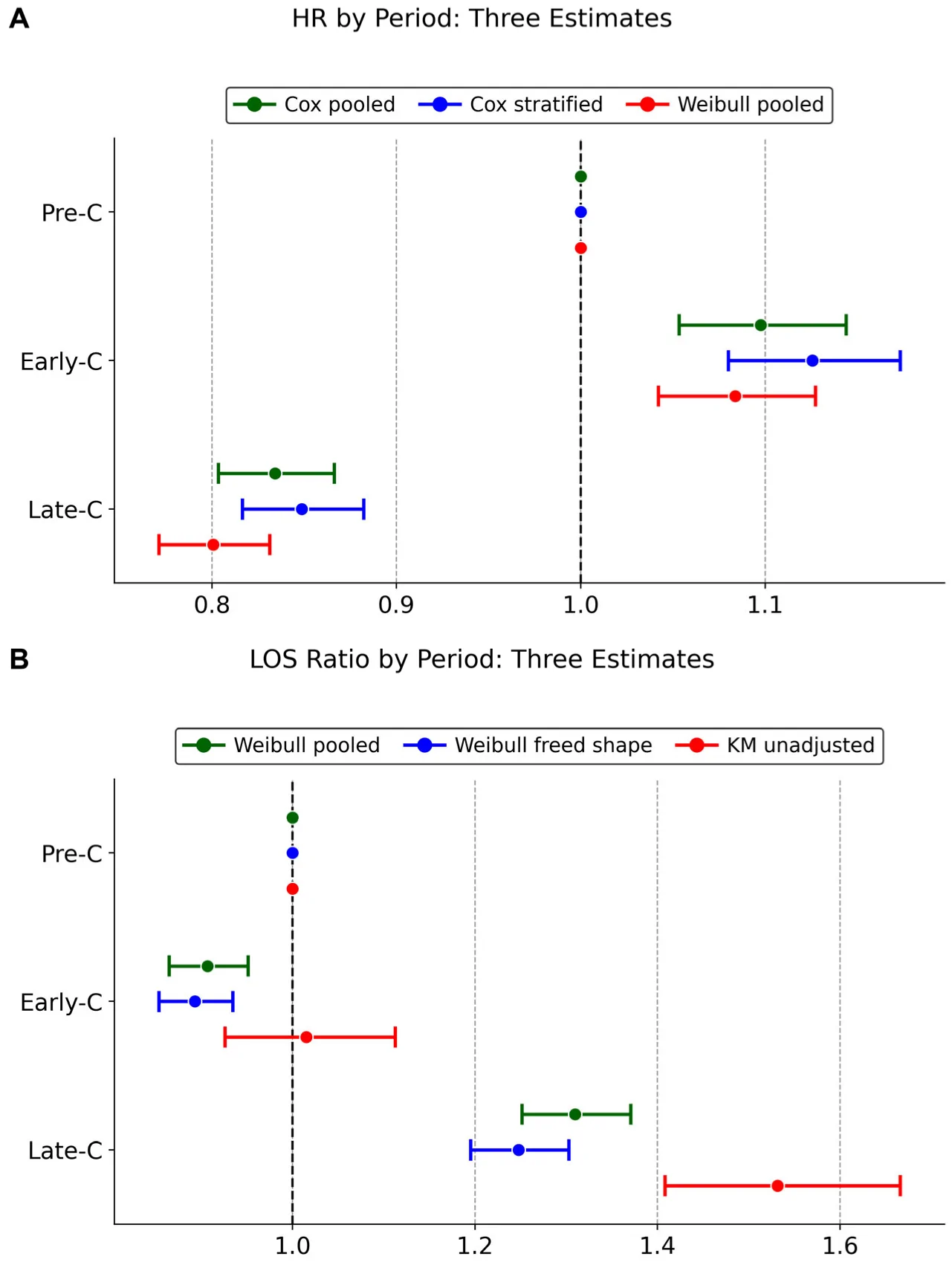
**The regression layer, by period, each estimate with its 95% confidence interval and the reference period at one.** (**A**) Hazard ratios, from the pooled Cox regression, from the per-factor stratified Cox fit, and implied by the pooled Weibull. (**B**) LOS ratios, from the pooled Weibull regression, the Weibull with the other factors’ shapes freed, and the unadjusted comparison of KM restricted means. Source files hazard_ratios_by_period and los_ratios_by_period, from the slide deck deposit [56].

**Table 1:** Length of stay and census by period, with 95% confidence intervals for the whole study window.

Period	Estimate	95% CI Lower	95% CI Upper
***Mean daily intakes***			
Pre-C	14.87	14.52	15.23
Early-C	8.81	8.54	9.09
Late-C	10.50	10.21	10.80
***Median LOS, days***			
Pre-C	6	6	6
Early-C	5	5	5
Late-C	6	6	7
***90th percentile LOS, days***			
Pre-C	25	23	28
Early-C	37	33	41
Late-C	54	50	60
***Restricted mean LOS, days***			
Pre-C	14.3	13.5	15.2
Early-C	14.5	13.5	15.5
Late-C	21.9	20.6	23.2
***Expected census, animals***			
Pre-C	212.8	199.7	226.7
Early-C	128.0	118.5	138.2
Late-C	230.2	215.4	246.0
***Fraction still in care at the cap***			
Pre-C	0.0034	0.0023	0.0052
Early-C	0.0019	0.0010	0.0034
Late-C	0.0042	0.0026	0.0070

Source is the results workbook [55] which arranges each estimate with its lower and upper bound vertically as three separate cells; here, each triplet has been transposed into a row with three columns.

**Table 2:** Workload by dog size, over the whole study window, averaged across days.

***Intake and care delivered over the study window***				
**Animal group**	**Days of care provided, whole window**	**Intakes/day**	**Intakes (%)**	
LARGE	179,474	4.4	38.4%	
MED	27,933	2.5	21.9%	
PUPPY	9053	1.2	10.4%	
SMALL	38,036	3.2	28.2%	
_UNKNOWN_	2863	0.1	1.0%	
***The population on an average day***				
**Animal group**	**Census, counted**	**Census counted (%)**	**Census, fitted**	**Census fitted (%)**
LARGE	131	69.7%	134	70.2%
MED	20	10.9%	20	10.7%
PUPPY	7	3.5%	7	3.5%
SMALL	28	14.8%	28	14.6%
_UNKNOWN_	2	1.1%	2	1.1%
***Tenure of that population, days***				
**Animal group**	**Tenure, counted**	**Tenure, fitted**	**Remaining, fitted**	
LARGE	68.1	70.1	71.1	
MED	11.3	11.3	12.3	
PUPPY	12.8	12.9	13.9	
SMALL	11.4	11.4	12.4	
_UNKNOWN_	26.5	26.7	27.7	
***Care of that population, animal-days***				
**Animal group**	**Animal-days, counted**	**Animal-days, fitted**	**Days owed, fitted**	**Days owed (%)**
LARGE	8924	9391	9525	92.7%
MED	231	231	251	2.4%
PUPPY	85	85	91	0.9%
SMALL	318	316	344	3.4%
_UNKNOWN_	55	56	59	0.6%

The source is one row-set of fourteen columns in the slide deck’s workbook [56], and the columns here respect the workbook’s order and headings but stack the table in sections. Counted values are read directly from the shelter data; fitted values are those expected at steady state from the KM curves and the observed intake rate.

## Data Availability

Software code is released under the MIT License. The user guide and the mathematical methods documentation are licensed under CC BY 4.0. A software deposit is cited by the DOI that resolves to the newest release, plus the version number. A data deposit is cited by its version DOI, because only the version record is guaranteed to hold the exact data used or results produced. Each software repository (mLOS and ShelterDataPrep) includes a machine-readable metadata file CITATION.cff. Raw animal-level extracts [58] from shelters including Orange County Animal Care, the input to the data preparation package: 10.5281/zenodo.22051091. ShelterDataPrep [21], the preparation package: 10.5281/zenodo.22051338, with version 0.3.0 the release that prepared the data used here; and 0.4.0 the current release as of this writing. Source GitHub repository: https://github.com/Shelter-Data-Analysis/ShelterDataPrep (accessed on 28 September 2026). Processed animal-level data [57], output of ShelterDataPrep and the direct input to mLOS: 10.5281/zenodo.22051368. These include descriptive statistics. mLOS [20] itself, the central software package: 10.5281/zenodo.22083814, with version 0.3.0 the release that produced the main results used here and also the current release as of this writing. Source GitHub repository: https://github.com/Shelter-Data-Analysis/mLOS (accessed on 28 September 2026). The main mLOS results (version 4) [55] for the worked example, at 10.5281/zenodo.23047014: the plots and the numerical values of the curves they draw, the results JSON, the Excel workbook, the console log, the preparation statistics, and the settings file that produced them. The experimental slide deck (version 5) [56] built from those results, together with the workbook of tables the build made: 10.5281/zenodo.23047087.

## Author Notes

An early version of this software generated material for a presentation at the American Board of Veterinary Practitioners 2026 Symposium (ABVP-2026) and a post-symposium extended abstract in JSMCAH [17]. Since then, mLOS capabilities (including in the form of the plots) evolved substantially. ShelterDataPrep and the OC2 data set it produces traceably from raw shelter data are a recent addition. The previous abstract example largely corresponds to the deposited OC1 dataset (except for minor filtering changes).

The software used in the earlier study published in PLOS One [11] and in the pre-symposium ABVP-2026 submission [10] is only a conceptual progenitor of mLOS. That initial software was in Python (rather than R), contained a narrower set of capabilities, and it relied on interactive execution. mLOS is not a port from Python to R but rather new code designed for broader use.

The AI system Claude Code Opus version 5 and earlier versions were extensively used in developing the software and its documentation. Almost all the code and the first draft of documentation were put together by AI. However, this was a multi-month process with many forks and iterations. There was extensive code testing, now built into the released software. The documentation was manually reviewed, edited, and adjusted in multiple iterations.

This paper was written by the author. The above Claude version was used to fetch information (including fragments of software documentation) relevant to each section of the paper. It was also used to review drafts and identify sections in need of refinement.
